# Effects of music-based interventions on cancer-related pain, fatigue, and distress: an overview of systematic reviews

**DOI:** 10.1007/s00520-023-07938-6

**Published:** 2023-07-24

**Authors:** Ana Trigueros-Murillo, Javier Martinez-Calderon, María Jesús Casuso-Holgado, Paula González-García, Alberto Marcos Heredia-Rizo

**Affiliations:** 1 Departamento de Fisioterapia, Facultad de Enfermería, Fisioterapia y Podología, Universidad de Sevilla, Seville, Spain; 2 CTS 1110: Uncertainty, Mindfulness, Self, and Spirituality (UMMS) research group, Andalusia, Spain; 3 Instituto de Biomedicina de Sevilla, IBiS, Departamento de Fisioterapia, Universidad de Sevilla, Seville, Spain

**Keywords:** Cancer, Fatigue: Meta-analysis, Music therapy, Pain, Systematic review

## Abstract

**Purpose:**

To summarize the available evidence from systematic reviews with meta-analysis on the effects of music-based interventions in adults diagnosed with cancer.

**Methods:**

An overview of systematic reviews was conducted. CINHAL, Embase, PEDro, PubMed, Scopus, the Cochrane Library and Web of Science were searched from inception until November 2022. Systematic reviews with meta-analysis in individuals with cancer (any type), any comparator, and outcomes of cancer-related pain, fatigue, and psychosocial symptoms were eligible. The methodological quality of systematic reviews and the amount of spin of information in the abstract were assessed. The Graphical Representation of Overlap for OVErviews tool (GROOVE) was used to explore the overlap of primary studies among systematic reviews.

**Results:**

Thirteen systematic reviews, with over 9000 participants, containing 119 randomized trials and 34 meta-analyses of interest, were included. Music-based interventions involved passive music listening or patients’ active engagement. Most systematic reviews lacked a comprehensive search strategy, did not assess the certainty in the evidence and discussed their findings without considering the risk of bias of primary studies. The degree of overlap was moderate (5.81%). Overall, combining music-based interventions and standard care seems to be more effective than standard care to reduce cancer-related pain, fatigue, and distress. Mixed findings were found for other psychosocial measures.

**Conclusion:**

Music-based interventions could be an interesting approach to modulate cancer-related pain, fatigue, and distress in adults with cancer. The variability among interventions, together with important methodological biases, detract from the clinical relevance of these findings.

**Supplementary Information:**

The online version contains supplementary material available at 10.1007/s00520-023-07938-6.

## Introduction

Nearly 18 million individuals are diagnosed with cancer every year [1]. Cancer is, therefore, a major cause of morbimortality and will continue to impose for long the highest clinical and socioeconomic disease-related burden worldwide for a long time [2]. Patients with cancer face physical impairments during and after treatment, often associated with increased levels of pain and fatigue [3, 4]. In addition, the complex and uncertain course of the disease [5] also leads to psychosocial challenges [6], i.e., anxiety and depression [7]. Cancer treatments usually focus on disease recurrence [8] and ongoing physical symptoms [7]. Yet, people with cancer now demand a more person-centered and comprehensive approach [9] that can address mental health problems [8].

Non-pharmacological therapies are of interest for the clinical management of long-term diseases, as considered to be safe, low-cost, and with minor side effects [10]. Among them, music-based interventions have shown to be useful in chronic conditions to improve the physical and emotional well-being in individuals with fibromyalgia [11] or affective disorders [12] and seem to help to modulate cancer-related symptoms [13–16]. Music-based interventions can be categorized as ‘music medicine’, i.e., passive listening of recorded music offered by healthcare staff, or ‘music therapy’ that encompasses the clinical use of music in all its forms, as provided by a credentialed therapist [16, 17]. Although both terms are often interchanged [18], a clear distinction is that music therapy involves individualized assessment, intervention, and evaluation, and a patient-therapist relationship that develops through the music [19]. Music-based interventions are characterized for using music in a passive or interactive modality (engaging a patient to create live music) and can be applied alone or within a multimodal program [16, 20–24]. Music is a highly structured language that engages complex cognitive, affective, sensory, and motor control processed in the human brain [25, 26]. Listening to music can reduce the activity of the autonomic nervous system, and improve the synchrony of the neural firing, which promotes brain plasticity [27]. Music can also appeal to strong emotional and social responses [28]. This provides a neural basis for the biological impact of music [29] and its influence on the physical and mental health[30]. Several systematic reviews have recently investigated the effectiveness of music-based interventions in cancer care [31, 32]. An overview of these systematic reviews can provide a high-level synthesis of evidence [33, 34]. It can also address the transparency of information and the methodological biases of previous research [34, 35], which may help to understand the clinical relevance of current evidence. The aim of this overview was to gather and assess the available evidence from systematic reviews with meta-analysis on the effectiveness of music-based interventions on physical and psychosocial outcomes in adults diagnosed with cancer.

## Methods

The overview protocol was prospectively registered at the Open Science Framework (https://doi.org/10.17605/OSF.IO/Y67BU). This overview has followed the preferred reporting items for overviews of reviews (PRIO) statement and the PRISMA for abstracts [36, 37]

### Deviations from intended protocol

There were no major deviations from the registered protocol.

### Search strategy

One researcher (ATM) carried out an electronic search from inception to November 2022 in the following databases: CINHAL, Embase, PEDro, PubMed, Scopus, the Cochrane Library, and Web of Science. Medical Subject Headings (MeSH) terms associated with the intervention (music) and the medical condition (e.g., cancer, neoplasm) were combined. A comprehensive search strategy was first constructed for PubMed and then adapted for other databases. The lists of references of previous overviews were manually checked. The detailed search strategies are listed as Supplementary file A.

### Eligibility criteria

The eligibility criteria were established following the PICOs framework (Population, Intervention, Comparison, Outcome, Study):

- P: Adults diagnosed with cancer without restrictions in body location/system or the cancer stage.
- I: music-based interventions, used alone or as adjuvant to usual or standard care
- C: no restrictions regarding the control intervention.
- O: physical (e.g., pain, fatigue), and psychosocial measures (e.g., anxiety, depression, mood, distress, and quality of life).
- S: systematic reviews with meta-analysis [38].

Systematic reviews were not included when: a) the publication was written in a language other than Spanish or English; b) there were not meta-analyses for the condition of interest; c) music-based interventions were meta-analyzed together with other experimental treatments; and d) meta-analyses included non-adult participants, population without cancer, or non-randomized controlled trials. Possible outcomes of interest that were not analyzed in at least two systematic reviews were not considered. Congress proceedings, thesis dissertations, and network meta-analyses were also excluded.

### Study selection

Duplicate records were removed using the Mendeley desktop software, v2.72.0. and manually checked. One researcher (ATM) screened the remaining records based on the title and the abstract. The full text of eligible studies and those lacking an abstract were then revised. A consensus was achieved for three studies with a second researcher (AMHR) who independently double-checked the entire selection process.

### Data extraction

Data were extracted with a standardized form that included: a) first author plus et al., the year of publication, and the number of clinical trials of interest; b) sample size (total and the experimental group); c) the characteristics of participants (age, sex, type of cancer); d) description of the experimental and control interventions; e) music style used; f) outcome measures; and g) main results from meta-analysis. We aimed to extract the overall effect size from each meta-analysis. When this was not reported, results from sub analyses were included. Two corresponding authors were contacted by e-mail to clarify some information [39, 40]. A reminder was sent, if necessary, one week after the first message. None of those contacted responded.

### Methodological quality

Two independent reviewers (ATM and MJCH) evaluated the methodological quality of systematic reviews using the AMSTAR-2 tool [41]. As recommended, individual ratings of the 16 items were not combined to obtain an overall score [42]. Instead, the attention was given to critical weakness domains, namely: item 2, prospective review protocol; item 4, comprehensive search strategy; item 7, justification of the excluded studies; item 9, risk of bias; item 11, appropriateness of statistical analysis; item 13, interpretation of results based on the risk of bias; and item 15, publication bias [42].

### Spin in abstracts of systematic reviews

The abstracts of the systematic reviews were assessed in isolation to quantify the occurrence of spin of information. Two independent reviewers (ATM and PGG) utilized a 7-item checklist [43], where each item was assigned a score of ‘yes’ or ‘no’.

### Data synthesis

Findings have been narratively described based on the outcomes of interest. To identify the most relevant key terms across systematic reviews, the VOSViewer software, v. 1.6.18 (Leiden University, The Netherlands) was used to conduct a co-occurrence analysis and bibliometric mapping. The degree of overlap of primary studies among included systematic reviews was evaluated with the Graphical Representation of Overlap for OVErviews (GROOVE)[44]. The GROOVE tool provides a simple, graphical and comprehensive representation, including the number of overlapped and non-overlapped primary studies and the overall assessment of the “Corrected Covered Area” (CCA), along with the CCA value for each pair of systematic reviews. For the CCA, the degree of overlap is considered to be slight (0–5%), moderate (6–10%), high (11–15%), and very high (CCA > 15%) [45]. Additionally, the CCA was measured taking into account chronological structural missingness, i.e., when primary studies were published after a systematic review [44].

## Results

**Fig. 1:**
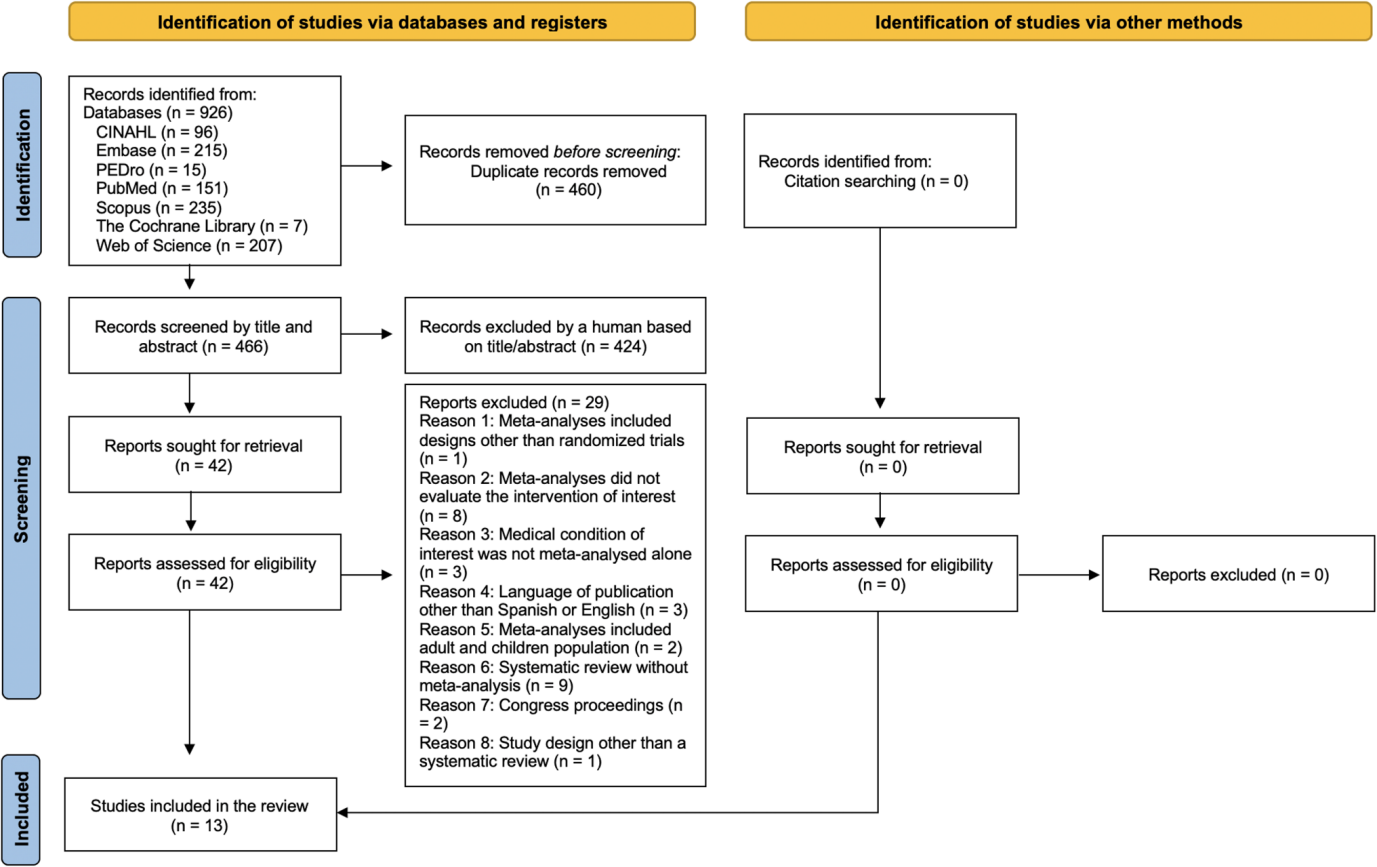
PRISMA flowchart

Search strategies retrieved a total of 926 eligible records. After removing duplicates, 466 records were screened. We eventually included 13 systematic reviews and 34 meta-analyses in the qualitative synthesis (Fig. 1). A list including the reports excluded during the final screening phase (*n* = 29) is described in the Supplementary file B.

Table 1 shows the characteristics of the included systematic reviews [39, 40, 46–56]. The most common types of cancer were breast and haematological, i.e., lymphoma and leukaemia. Music-based interventions were often combined with specific cancer treatments (e.g., surgery, chemotherapy or radiotherapy) or with standard or usual care, and involved passive listening of live or recorded music or patient’s active engagement (e.g., singing, clapping, and guided music imagery). Different music styles, selected by therapists or patients’ preferences, were used. A 23% of systematic reviews judged the overall certainty in the evidence using the Grading of Recommendations Assessment, Development and Evaluation (GRADE) approach [49, 55, 56]. Most reviews (77%) assessed the risk of bias of the clinical trials using the Cochrane Risk of Bias tool.

**Table 1:** Characteristics of the included systematic reviews

Study; RCTs of interest	Participants (age, sex); diagnosis	Interventions, duration	Music style	Outcomes	Effect size (overall or subgroup analysis)
Bradt et al. 2021; Australia (1), Brazil (5), China (13), Denmark (2), Germany (1), Iran (3), Italy (3), Taiwan (4), Turkey (1), USA (36) Total: 69	Total: 4838 (54.8 yrs., 3015 females); music-based group (2900) Breast, haematological (lymphoma, leukaemia, myeloma), lung, rectal, skin, neck, head, sinus, genitourinary, and others	Music-based group: standard care with live or recorded music). Active involvement, i.e., singing, imagery, clapping, or passive listening. Applied during radio or chemotherapy, surgery, or other Control group: standard care, i.e., chemo or radio therapy or surgery, alone or combined with wearing headphones with no music Sessions: 1 to 40 Duration: 1 day to 15 wks	Blues, pop, rock, country, new age, classical	Anxiety: STAI, NRS, HADS, QMS, SAS, VAS Depression: BDI, HADS, CES-D, POMS, QMS, SDS Distress: NRS Fatigue: BFI, MSFI, VAS, FACIT, POMS, EORTC Mood: SF-POMS, VAS, POMS, EORTC Pain: BPI, NRS, VAS QoL: HQLI-R, FACIT-G, EORTC, FACIT-BMT	• Anxiety (subgroup, STAI, 17 studies) **SMD: -7.73 (-10.02, -5.44), *P* < 0.01, I**^**2**^** = 93%** • Anxiety (subgroup, non-STAI, only cancer, 8 studies) **SMD: -0.75 (-1.30, -0.21), *P* = 0.007, I**^**2**^** = 92%** • Depression (subgroup, only cancer, 11 studies) **SMD: -0.41 (-0.68, -0.15),*P* = 0.002, I**^**2**^** = 75%** • Distress (overall, 2 studies) SMD: -0.38 (-1.43, 0.66), P = 0.47, I^2^ = 88% • Fatigue (subgroup, only cancer, 9 studies) **SMD: -0.26 (-0.44, -0.07), *P* = 0.007, I**^**2**^** = 0%** • Mood (overall, 4 studies) SMD: 0.53 (-0.03, 1.10); *P* = 0.07; I^2^ = 76% • Pain (subgroup, only cancer, 9 studies) **SMD: -0.77 (-1.25, -0.29), *P* = 0.002, I**^**2**^** = 85%** • QoL (overall, 7 studies) **SMD: 0.88 (-0.31, 2.08), *P* = 0.015, I**^**2**^** = 97%**
Bro et al. 2018; China (3), Germany (1), Iran (1), Italy (2), Taiwan (2), Turkey (1), USA (15) Total: 25	Total: 1784 (age, sex, N/S); music-based group (N/S) Breast, haematological (lymphoma, leukaemia) or N/S	Music-based group: standard care and live or recorded music. Active involvement, i.e., singing, imagery, clapping, or passive listening. Applied during radio or chemotherapy, surgery, or other Control group: standard care, wait-list Sessions: 1 to 45 Duration: 3 days to 60 wks	Classical, rock and roll, folk, new age, jazz, soundtracks, nature music, religious	Anxiety: HADS, STAI, VAS Depression: HADS, ZSDS Distress: HADS, NRS Fatigue: BFI, POMS, VAS Mood: I-PANAS-SF, VAS, SF-POMS Pain: MPQ, NRS, VAS QoL: EORTCBR23/ QLQ‐30, FACIT‐G	• Anxiety (overall, 9 studies) **SMD: -0.65 (-1.20, -0.11), *P* = 0.02, I**^**2**^** = 90%** • Depression (overall, 2 studies) SMD: -0.89 (-2.92, 1.14), *P* = 0.39, I^2^ = 97% • Distress (overall, 2 studies) SMD: -0.25 (-1.03, 0.52); *P* = 0.52, I^2^ = 74% • Fatigue (overall, 3 studies) SMD: -0.22 (-1.08, 0.63), *P* = 0.61, I^2^ = 80% • Mood (overall, 4 studies) **SMD: -0.55 (-0.98, -0.13), *P* = 0.01, I**^**2**^** = 54%** • Pain (overall, 7 studies) **SMD: -0.88 (-1.45, -0.32), ***P*** = 0.002, I**^**2**^** = 88%** • QoL (overall, 2 studies) SMD: -0.21 (-0.55, 0.14), *P* = 0.25, I^2^ = 0%
Garza-Villarreal et al. 2017; USA (1), Italy (1), China (1), Taiwan (1) Total: 4	Total: 361 (55.3 yrs., 223 females); music-based group (N/S) Diagnosis N/S	Music-based group: live or recorded music Control group: standard care. Sessions: N/S Duration: N/S	Music style: N/S	Pain: NRS, VAS,	• Pain (overall, 4 studies) **SMD: -0.78 (-1.00, -0.56), *P* < 0.001, I**^**2**^** = 71%**
Nguyen et al. 2022; Brazil (1), Italy (1), Germany (2), Taiwan (2), USA (3) Total: 9	Total: 686 (age, sex, N/S); music-based group (381) Breast, haematological (lymphoma, leukaemia) or mixed	Music-based group: standard care with music therapy or music medicine. Recorded or live music. Active involvement, i.e., singing, playing, guided imagery, or passive listening. Applied before or during chemotherapy or at hospital Control group: standard care Sessions: 1 to 36 Duration: 1 day to 12 wks	Classical, new age, blues, jazz, pop, rock, folk, nature sounds, film soundtracks	Anxiety: HADS, STAI VAS Depression: BDI-II, HADS QoL: QLQ‐30, WHOQOL	• Anxiety (overall, 6 studies) **SMD: -0.29 (-0.50, -0.08), *P* = 0.006; I**^**2**^** = 62%** • Depression (overall, 3 studies) SMD: -0.04 (-0.32, 0.24), *P* = 0.79; I^2^ = 0% • QoL (overall, 2 studies) **SMD: 0.42 (0.02, 0.82), *P* = 0.04, I**^**2**^** = 18%**
Nightingale et al. 2013; China (1), Italy (1), Taiwan (1), USA (10) Total: 13	Total: 709 (51 to 63 yrs., sex N/S); music-based group (383) Breast, haematological (lymphoma, leukaemia) lung, prostate, head and neck, colorectal, stomach, ovarian, skin, others, or N/S	Music-based group: treatment as usual care with recorded or live music. Active involvement, i.e., singing, playing, or passive listening. Applied at hospital stay or during treatment Control group: treatment as usual care alone or combined with headphones with no music Sessions: 1 to 15 Duration: 1 day to 5 wks	Classical, soundtracks, nature, new age, country, religious or other	Anxiety: BAI, HADS, VAS, POMS-SF, ZSAS, STAI	• Anxiety (overall, 4 studies) SMD: -0.003 (-0.51, 0.52), *P* = 0.99, I^2^ = 67.5%
Park et al. 2021; China (1), Italy (1), Taiwan (1) Total: 3	Total: 301 (54.4 yrs., sex N/S); music-based group (151) Breast or mixed	Music-based group: standard care with recorded or live music Control group: usual care Sessions: 1 to 4 or tailored Duration: 1 day to 4 wks	Sedative music or for relaxation	Pain: SF-MPQ, VAS	• Pain (overall, 3 studies) **Hedges’ g: 0.86 (0.53, 1.18), *P* < 0.01, I**^**2**^** = 37%**
Qi et al. 2021; China (3), Italy (1), USA (4) Total: 8	Total: 467 (53.2 yrs., sex N/S); music-based group (235) Breast, haematological (lymphoma, leukaemia, myeloma), or N/S	Music-based group: standard care with recorded or live music. HELP mode music therapy, FEMT Control group: standard care, relaxation training, or N/S Sessions: 1 to 40 Duration: 1 day to 8 wks	Music style: N/S	Fatigue: BFI, POMS, SF-MFSI, SF-POMS, VAS	• Fatigue (overall, 8 studies) **SMD: -0.88 (-1.49, -0.26), *P* = 0.005, I**^**2**^** = 89%**
Sezgin and Bektas 2022; USA (6) Total: 6	Total: 279 (56 yrs., sex N/S); music-based group (140) Haematological (lymphoma, leukaemia, myeloma)	Music-based group: standard care with passive music listening (with or without imagery) Control group: standard care Sessions: 1 to 8 Duration: 1 day to 4 wks	Guitar, classical, others	Fatigue: BFI, FACIT-F, POMS, VAS	• Fatigue (overall, 6 studies) **Hedges’ g: 0.33 (0.09, 0.56), *P* = 0.006, I**^**2**^** = 0%**
Tao et al. 2016; China (3) Total: 3	Total: 300 (age, sex N/S); music-based group (N/S) Mixed or breast	Music-based group: usual or standard medical care and FEMT or FEMT + placebo Control group: usual or standard medical care Sessions: N/S Duration: N/S to 12 wks	Chinese Medicine FEMT	Depression: SDS	• Depression (overall, 2 studies) **SMD: -2.96 (-5.60, -0.32), *P* = 0.03, I**^**2**^** = 96%**
Tsai et al. 2014; Country N/S Total: 18	Total: 1405 (51.7 yrs., sex N/S); music-based group (665) Breast, haematological, (lymphoma, leukaemia) colorectal, or N/S	Music-based group: usual or standard care with passive music listening or active involvement Control group: usual or standard care Sessions: 1 to 10 Duration: 1 to 4 wks	N/S	Depression: HADS, POMS, ZSDS Fatigue: FACIT-F, POMS Pain: NRS, VAS, SF-MPQ	• Depression (subgroup, adults, 7 studies) **Hedge’s g: -0.55 (-0.73, -0.37), *P* = 0.001, I**^**2**^** = N/S** • Fatigue (subgroup, adults, 5 studies) **Hedge’s g: -0.49 (-0.73, -0.24), *P* = 0.001, I**^**2**^** = 28.52%** • Pain (subgroup, adults, 4 studies) **Hedge’s g: -0.58 (-1.05, -0.11), *P*** = 0.016, I^**2**^** = N/S**
Wang et al. 2018; Country N/S Total: 30	Total: 2559 (18 to 75 yrs., sex N/S); music-based group (1292) Breast	Music-based group: passive listening alone or with imagery and relaxation. Applied before or during radiotherapy or surgery Control group: standard care Sessions: 1 to 180 Duration: 2 days to 18 wks	Music style: N/S	Anxiety: HARS, SAI, SAS Depression: SDS	• Anxiety (HARS, 2 studies) **MD: -7.04 (-9.31, -4.78), *P* < 0.001, I**^**2**^** = 87%** • Anxiety (SAI, 2 studies) **MD: -12.40 (-21.86, -2.94), *P* = 0.01, I**^**2**^** = 64%** • Anxiety (SAS, 5 studies) **MD: -7.40 (-10.28, -4.52), *P* < 0.001, I**^**2**^** = 90%** • Depression (SDS, 6 studies) **MD: -7.39 (-8.35, -6.43), *P* < 0.001, I**^**2**^** = 31%**
Yang et al. 2021; China (13) Total: 13	Total: 1174 (51.9 yrs., 352 females); music-based group (650) Breast, colorectal, leukaemia, lung, others	Music-based group: usual care with FEMT Control group: usual care, massage, relaxation training Sessions: 6 to 180 Duration: 3 days to 12 wks	Music style: Chinese Medicine FEMT	Anxiety: SAS Depression: SDS	• Anxiety (SAS, 7 studies) SMD: -0.45 (-1.59, 0.70), P = 0.45, I^2^ = 97% • Depression (SDS, 10 studies) **SMD: -1.34 (-1.69, -0.98), *P* < 0.001, I**^**2**^** = 80%**
Yangöz et al. 2019; Brazil (1), Italy (2), Taiwan (1), Turkey (1), USA (1) Total: 6	Total: 593 (age, sex, N/S); music-based group (279) Diagnosis N/S	Music-based group: standard care with passive music listening (live or recorded music) Control group: standard care, bed rest Sessions: 1 to 3 Duration: 1 day to 4 wks	Instrumental, pop, jazz, classical, soundtracks, piano, or N/S	Pain: ESAS, NRS, VAS	• Pain (overall, 6 studies) **Hedges’ g: 0.56 (0.19, 0.92), *P* = 0.003, I**^**2**^** = 76.83%**

Effect size data are reported as mean difference, or standard mean difference with (95% confidence interval). I^2^ index indicates the level of heterogeneity. Bold data indicates statistically significant differences (*p*<0,05). Subgroup analyses are listed when an overall meta-analysis was not available. Age and sex data are reported when included in more than half of the studies

Abbreviations: *BAI* Beck Anxiety Inventory, *BDI* Beck Depression Inventory, *BFI* Brief Fatigue Inventory, *BPI* Brief Pain Inventory, *CES-D* Center for Epidemiologic Diseases – Depression Scale, *EORTC* European Organization for Research and Treatment on Cancer, *EORTC-BR23* European Organization for Research and Treatment for Breast Cancer Women, *EORTC-QLQ-30*, *FACIT-BMT* Functional Assessment of Chronic Illness Therapy-Bone Marrow Transplant, *ESAS* Edmonton Symptom Assessment System, *FACIT-G* Functional Assessment of Chronic Illness Therapy-Fatigue, *FACIT-G* Functional Assessment of Cancer Therapy-General, *FEMT* Five-Elements Music Therapy, *HADS* Hospital Anxiety and Depression Scale, *HARS* Hamilton Anxiety-Rating Scale, *HQLI-R* Hospice Quality of Life Index – Revised, *I-PANAS-SF* positive and negative affect schedule-short form, *MD* mean difference, *MFSI* Multidimensional Fatigue Inventory, *MPQ* Mc Gill Pain Questionnaire, *N/S* non-specified, *NRS* Numeric Rating Scale, *POMS* Profile of Mood Sates, *QLQ‐30* Quality of Life questionnaire, *QMS* Quick Mood Scale, *QoL* quality of life, *RCT* randomized controlled trial, *SAI* State Anxiety Inventory, *SAS* State Anxiety Scale, *SDS* Self-Rating Depression Scale, *SF* short-form, *SMD* standard mean difference, *STAI* Spielberger State-Trait Anxiety Inventory, *VAS* Visual Analogue Scale, *wks.* Weeks, *WHOQOL* World Health Organization Quality of Life, *yrs.* Years, *ZSAS* self-rating anxiety scale, *ZSDS* self-rating depression scale

### Methodological quality

Results for the AMSTAR 2 tool are described in Table 2 (inter-rater agreement, 78.8%). The most important methodological concerns were ‘the lack of comprehensive search strategies’, ‘no information of the excluded studies’, and the ‘interpretation of the review findings without accounting for the risk of bias of primary research’. More than 90% of systematic reviews did not inform of why they included a certain type of study design or about their funding sources.

**Table 2:**
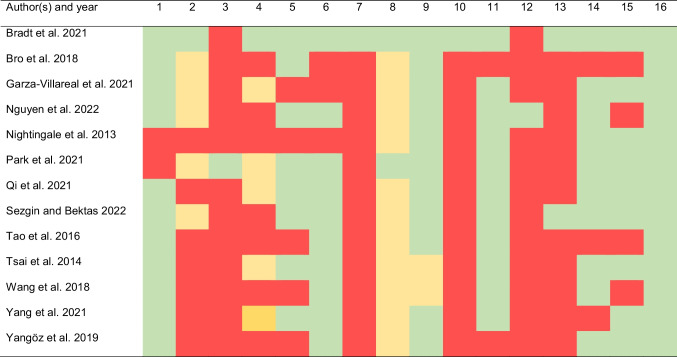
Risk of bias (AMSTAR 2) of the included systematic reviews

Abbreviations: *AMSTAR* A MeaSurement Tool to Assess systematic Reviews. Green color = yes; Red color = no; Orange color = Partial yes

AMSTAR 1: Did the research questions and inclusion criteria for the review include the components of PICO? AMSTAR 2: Did the report of the review contain an explicit statement that the review methods were established prior to the conduct of the review and did the report justify any significant deviations from the protocol? AMSTAR 3: Did the review authors explain their selection of the study designs for inclusion in the review? AMSTAR 4: Did the review authors use a comprehensive literature search strategy? AMSTAR 5: Did the review authors perform study selection in duplicate? AMSTAR 6: Did the review authors perform data extraction in duplicate? AMSTAR 7: Did the review authors provide a list of excluded studies and justify the exclusions? AMSTAR 8: Did the review authors describe the included studies in adequate detail? AMSTAR 9: Did the review authors use a satisfactory technique for assessing the risk of bias in individual studies that were included in the review? AMSTAR 10: Did the review authors report on the sources of funding for the studies included in the review? AMSTAR 11: If meta-analysis was performed did the review authors use appropriate methods for statistical combination of results? AMSTAR 12: If meta-analysis was performed, did the review authors assess the potential impact of risk of bias in individual studies on the results of the meta-analysis or other evidence synthesis? AMSTAR 13: Did the review authors account for risk of bias in individual studies when interpreting/ discussing the results of the review? AMSTAR 14: Did the review authors provide a satisfactory explanation for, and discussion of, any heterogeneity observed in the results of the review? AMSTAR 15: If they performed quantitative synthesis did the review authors carry out an adequate investigation of publication bias (small study bias) and discuss its likely impact on the results of the review? AMSTAR 16: Did the review authors report any potential sources of conflict of interest, including any funding they received for conducting the review?

### Spin of information in abstracts

The overall spin-abstract score was 21, with a mean value of 1.6 ± 1.3 points (inter-rater agreement, 79%). The most common forms of spin were ‘concluding a positive effect despite high risk of bias of primary trials’ (*n* = 7), and ‘selective reporting or overemphasis on the beneficial effect of music-based intervention’ (*n* = 5). No spin of information was found in three abstracts [46, 47, 52] (Supplementary file C).

### Co-ocurrence analysis

**Fig. 2:**
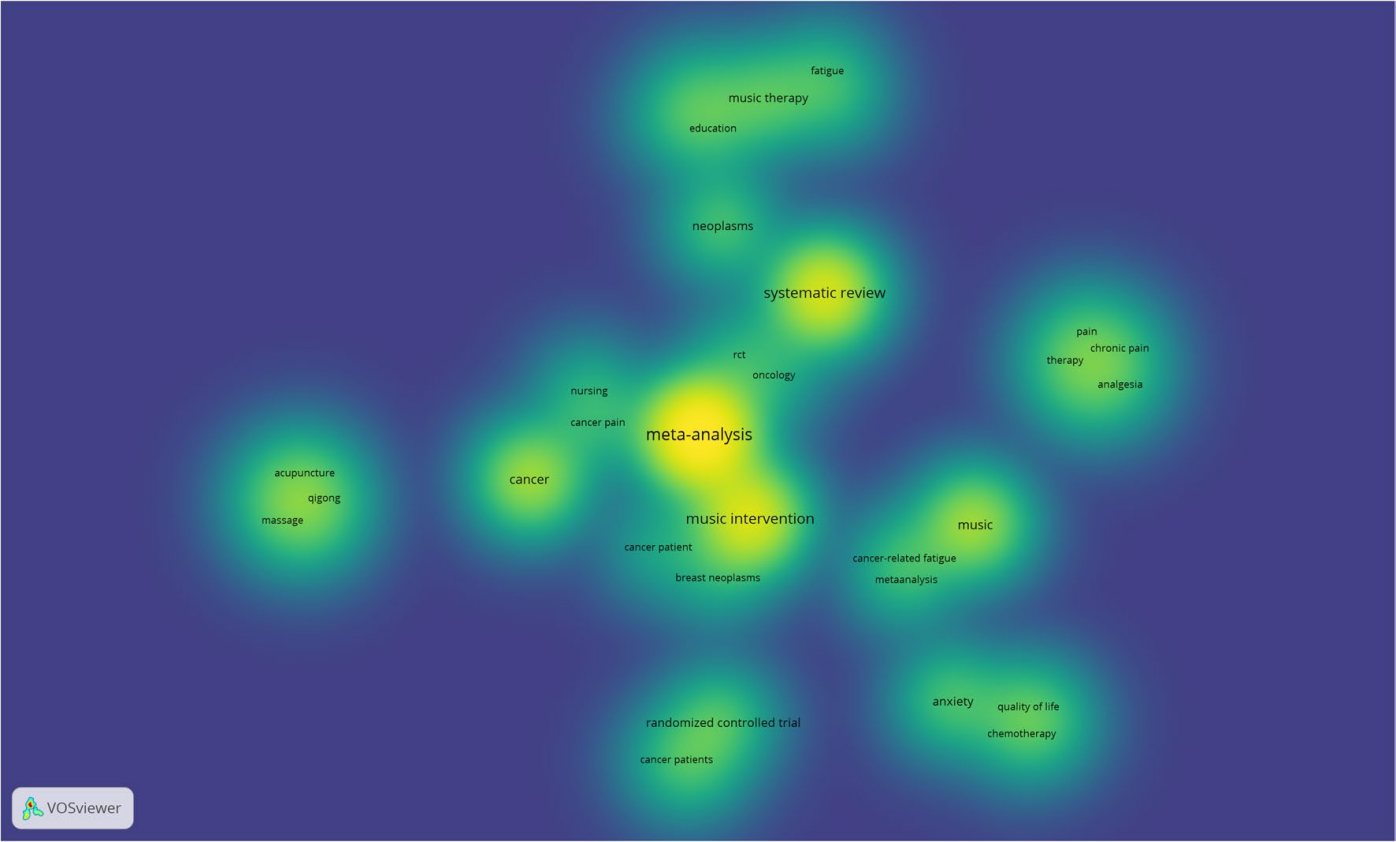
Density visualization

**Fig. 3:**
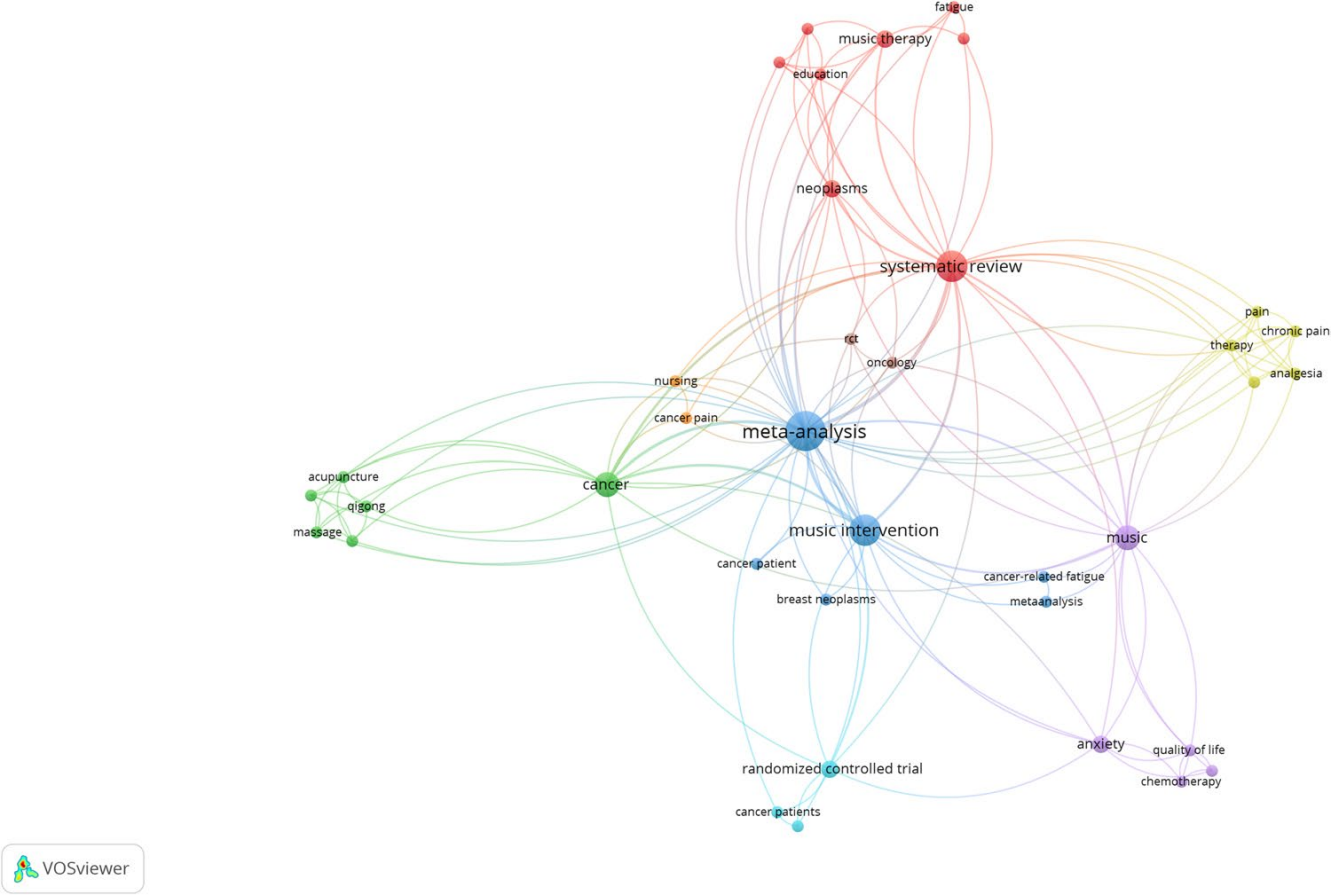
Network visualization

Twelve out of the thirteen systematic reviews were included in the co-occurrence analysis (Figs. 2 and 3). One review did not include key terms [56]. The pattern of association between keywords has been reflected in the network and density visualizations The terms most frequently used were related to the research design (meta-analysis, systematic review), the intervention (music interventions, music), and the disease (cancer, neoplasms).

### Overlapping between primary study

A total of 202 primary studies were identified across the included systematic reviews, out of which 119 were distinct studies. The overall overlap for the entire matrix of evidence was moderate (CCA = 5,81%) and this remained moderate (CCA = 6,92%) even after adjusting for chronological structural missingness. The citation matrix and the CCA calculation can be found in Supplementary file D. The Supplementary file E presents the graphical representation of the GROOVE tool. Three primary studies from one of the systematic reviews could not be retrieved due to the insufficient information and a lack of response from the corresponding author [39].

#### Music-based interventions on cancer-related pain

All systematic reviews measuring pain as an outcome (*n* = 6) concluded that music-based interventions plus usual or standard care were more effective than control interventions (e.g., usual or standard care, wait-list, bed rest, or wearing headphones with no music) to reduce cancer-related pain [39, 46, 48, 49, 52, 56].

#### Music-based interventions on cancer-related fatigue

Among the five systematic reviews assessing cancer-related fatigue, four of them indicated that combining music-based interventions with usual or standard care could yield more benefits than control interventions to improve cancer-related fatigue [39, 50, 54, 56]. However, one systematic review found no differences between groups [49].

#### Music-based interventions on cancer-related anxiety

Inconclusive conclusions were detected upon evaluating the six systematic reviews assessing cancer-related anxiety [40, 47, 49, 51, 55, 56].

#### Music-based interventions on cancer-related depression

Inconclusive conclusions were detected upon evaluating the seven systematic reviews investigating cancer-related depression [39, 40, 49, 51, 53, 55, 56].

#### Music-based interventions on cancer-related mood and distress

Two systematic reviews concluded that music-based interventions together with usual or standard care could be more effective than controls in reducing cancer-related distress [49, 56]. However, findings on patients’ mood varied across studies [49, 56].

#### Music-based interventions on cancer-related quality of life

Two out of the three systematic reviews on quality of life demonstrated that music-based interventions combined with usual or standard care were superior to usual care alone in improving health-related quality of life [55, 56], while one review found no significant differences between groups [49].

### Adverse events of music-based interventions

Four systematic reviews provided information regarding potential adverse events. In all of these reviews, no adverse events were observed following music-based interventions [46, 50, 52, 56].

## Discussion

This overview summarized the evidence from systematic reviews with meta-analysis on the effects of music-based interventions to modulate cancer-related symptoms in adults. Overall, our results seem to suggest that adding music interventions to usual or standard care could be more beneficial than usual care alone to reduce cancer-related pain, fatigue, and distress. On the other hand, findings were inconclusive for anxiety, depression, mood, and quality of life.

The present results expand those of previous overviews underlying the importance of including music-based within a multimodal treatment to decrease cancer-related pain [33, 57]. However, this is the first overview specifically focused on music-based interventions. Music involves cognitive engagement and distraction [58, 59]. Listening to music can help to the release of endogenous opioids and dopamine [58], which supports music-induced analgesia and may contribute to reduce the severity of fatigue [54]. Cancer-related pain is a complex, evolving, and multifaceted phenomenon [60], comprised of several dimensions (sensory. discriminatory, emotional, cognitive, and behavioral) [61]. Complementary integrative therapies, such as music-based interventions, can effectively manage cancer pain [61]. However, music may exert distinct influences on the different dimensions of pain, thus contributing to divergent findings observed in both physical and psychological measures. The impact of music on cancer-related fatigue has found to be highly relevant when music is combined with other therapies, e.g., exercise, especially when the intervention involves active patient’s engagement [50]

Current clinical practice guidelines recommend the use of music to manage the cancer-related psychological burden during and after treatment [62, 63]. However, the exact mechanisms to understand how music engagement contributes to mental health remain unknown [64]. We found inconclusive results for anxiety, depression, mood, and the quality of life. This is in line with prior findings reported in palliative cancer care [65], but contradicts those for non-adult cancer populations [66, 67]. This might be because children and adolescents with cancer do not have as many comorbidities as adults and often tend to respond better to treatment. The style of music, along with personality, cultural, and contextual factors have an influence on the effects of music [64, 68]. Also, the diversity of tools used to measure anxiety and depression may contribute to the inconsistency of results [68]. In summary, more definite conclusions could be drawn with less heterogeneity in participants’ characteristics, especially age and cancer stage, assessment tools, and music intervention protocols.

### Clinical relevance

This overview provides an updated synthesis of evidence about the use of music as an adjuvant therapy for adults in cancer care. Given that music is a potential cost-effective intervention [58], the present findings seem to encourage clinicians to implement its use into daily practice. There are, however, barriers that need to be overcome, mostly related to the lack of practical guidelines for music dose and timing [69]. Researchers have a strong responsibility to provide a complete description of interventions. That is the sole purpose of the TIDieR checklist, designed to advance evidence-based clinical practice [70]. However, none of the included systematic review provided information about how well described music-based interventions were in primary trials, which detracts from their replicability. Other important aspects should be born in mind. First, a clear distinction between music medicine or music therapy can be clinically relevant but it was only made in two of the systematic reviews [55, 56]. Music therapy was superior to music medicine to improve the quality of life and fatigue [56], but worse than music medicine for reducing anxiety [55]. These results suggest that the person who conducts the intervention and the mode of delivery may be clinically relevant. Second, treatment benefits following music interventions may depend on patients’ characteristics, e.g., emotional vulnerability [71]. Third, the lack of adverse effects suggests that music is a safe intervention in this population, although information regarding potential adverse events was only reported in four systematic reviews [46, 50, 52, 56]. Finally, most systematic reviews did not clarify whether ‘standard’ or ‘usual’ care included supportive cancer care, as a paradigm for modern treatment in oncology [72], to manage the physical, psychological, social, and spiritual needs of patients [73], or specific cancer treatments such as chemotherapy. This needs to be clarified in future systematic reviews.

### Methodological concerns

We have addressed, for the first time, potential biases, and transparency of information of systematic reviews in this field. The main concerns were related to the search strategy and the interpretation of the results without accounting for potential risk of bias. This may lead to selection bias and to an inaccurate translation of the findings to the clinical setting. It is somehow concerning that 40% of the reviews ‘overemphasized’ the beneficial impact of the music intervention group. Unfortunately, this misleading presentation of results is not new in the context of cancer treatment [74]. The certainty in the evidence using the GRADE framework was only evaluated in three systematic reviews [49, 55, 56]. In addition, as previously stated, the presence of adverse events, which is highly relevant, was poorly reported. Both aspects need to be carefully considered to improve the standard of quality. We incorporated chronological structural missingness to calculate the degree of overlap with the GROOVE tool, which is a novel and interesting approach. The GROOVE may also enable the analysis of overlap for specific outcomes, but this feature was not considered due to the high heterogeneity of measurement tools among the included reviews. Finally, evidence from clinical trials need to be complemented by qualitative studies to get a whole idea of music as individualized therapy.

### Limitations

Literature search screening was conducted by a single researcher. Congress proceedings, network meta-analysis and reviews not written in Spanish or English were excluded, thus meaningful information may have been overlooked. The PICOs question considered music-based interventions in general and was not limited to music therapy or music medicine. In addition, the overlap of clinical trials among reviews prevented us to conduct meta-meta-analysis or to evaluate the certainty in the evidence.

## Conclusions

Based on our results, we can conclude that:

- The combination of music-based interventions with standard or usual care could be more effective than standard care alone to reduce cancer-related pain, fatigue, and distress in adults diagnosed with cancer.
- The additive effect of music-based interventions to standard or usual care remains uncertain for anxiety, depression, mood, and the quality of life.
- Clinical and methodological concerns have been discussed and should be carefully considered when interpreting our findings in a clinical context.

### Supplementary Information

Below is the link to the electronic supplementary material.

Supplementary file1 (DOCX 20 KB)

Supplementary file2 (DOCX 36 KB)

Supplementary file3 (DOCX 15 KB)

Supplementary file4 (DOCX 52 KB)

Supplementary file5 (DOCX 18 KB)

Supplementary file6 (DOCX 25 KB)

## Data Availability

The data that support the study findings are available from the corresponding author upon request.
