# Correction to: Effects of music-based interventions on cancer-related pain, fatigue, and distress: an overview of systematic reviews

**DOI:** 10.1007/s00520-024-08478-3

**Published:** 2024-04-05

**Authors:** Ana Trigueros-Murillo, Javier Martinez-Calderon, María Jesús Casuso-Holgado, Paula González-García, Alberto Marcos Heredia-Rizo

**Affiliations:** 1 Departamento de Fisioterapia, Facultad de Enfermería, Fisioterapia y Podología, Universidad de Sevilla, Seville, Spain; 2 CTS 1110: Uncertainty, Mindfulness, Self, and Spirituality (UMMS) Research Group, Andalusia, Spain; 3 Instituto de Biomedicina de Sevilla, IBiS, Departamento de Fisioterapia, Universidad de Sevilla, Seville, Spain

**Correction to: Supportive Care in Cancer (2023) 31:488**

10.1007/s00520-023-07938-6

On behalf of all authors, we regret to inform you that an error has been identified in the aforementioned article regarding the reported results. Throughout the text, the results indicate that the studied intervention has been effective in reducing pain, fatigue, and distress in subjects with cancer. However, upon review, it has come to our attention that the reviews included did not find any benefit of music-based intervention in reducing distress (the data can be seen in Table 1).

Therefore, in statements where the effectiveness of the intervention is granted for

- “pain, fatigue, ***and distress***,”

it should instead be substituted to

- “pain, fatigue, ***but not distress***.”

These sentences can be read in the abstract (page 1: last sentence of results and first of conclusions), in the discussion (page 9: last sentence of the first paragraph) and in conclusions (page 11: first bullet point).

Additionally, in the analysis of the results on different outcomes, in “Music-based interventions on cancer-related mood and distress” (page 8) where it is written:

- “Two systematic reviews concluded that music-based interventions together with usual or standard care could be more effective than controls in reducing cancer-related distress”, the words “could be” must be substituted by “were not”.

We sincerely apologize for any inconvenience this may have caused to the Editors, the journal, and the readers.

