# Supplementary material for: Effects of music-based interventions on cancer-related pain, fatigue, and distress: an overview of systematic reviews
Source: Support Care Cancer. 2023 Jul 24;31(8):488. doi: 10.1007/s00520-023-07938-6 (PMC10366242; doi:10.1007/s00520-023-07938-6)
Supplement: Supplementary file 1 — Supplementary file1 (DOCX 20 KB) [file 520_2023_7938_MOESM1_ESM.docx]

**Supplementary File A.** Search Strategies

1. **CINAHL (date November 21, 2022)**

(music* OR "music therapy" OR sing OR song* OR rhythm*) AND (cancer OR tumo* OR neoplas* OR oncolog* OR malignan*) AND (meta review OR systematic review OR meta-analys*)

**(studies retrieved = 96)**

1. **Embase (date November 21, 2022)**

((music* OR "music therapy" OR sing OR song* OR rhythm*) AND (cancer OR tumo* OR neoplas* OR oncolog* OR malignan*) AND (‘systematic review’:ti OR ‘meta review’:ti OR ‘meta analys*’:ti))

**(studies retrieved = 215)**

1. **PEDro (date November 21, 2022)**

cancer AND music

**(studies retrieved = 15)**

1. **PubMed (date November 21, 2022)**

(music*[tiab]) OR "music Therapy"[tiab] OR sing[tiab] OR song*[tiab] OR rhythm*[tiab]) AND (cancer[tiab] OR tumo*[tiab] OR neoplas*[tiab] OR oncolog*[tiab] OR malignan*[tiab]) AND (systematic review [tiab] OR meta analys* [tiab] OR meta review [tiab])

**(studies retrieved = 151)**

1. **Scopus (date November 21, 2022)**

(music* OR "music therapy" OR sing OR song* OR rhythm*) AND (cancer OR tumo* OR neoplas* OR oncolog* OR malignan*) AND (meta review OR systematic review OR meta-analys*)

**(studies retrieved = 235)**

1. **The Cochrane Library (date November 21, 2022)**

#1 (“Systematic review”):ti,ab,kw

#2 (“meta-analys*”):ti,ab,kw

#3 (“meta review”):ti,ab,kw

#4 (music*)

#5 (“music therapy”)

#6 (sing)

#7 (song*)

#8 (rhythm*)

#9 (cancer)

#10 (tumo*)

#11 (neoplas*)

#12 (oncolog*)

#13 (malignan*)

#14 ((#1 OR #2 OR #3) AND (#4 OR #5 OR #6 OR #7 OR #8) AND (#9 OR #10 OR #11 OR #12 OR #13))

**(Cochrane reviews retrieved = 7)**

1. **Web of Science (date November 21, 2022)**

(music* OR "music therapy" OR sing OR song* OR rhythm*) AND (cancer OR tumo* OR neoplas* OR oncolog* OR malignan*) AND (meta review OR systematic review OR meta-analys*)

**(studies retrieved = 207)**
