# Supplementary material for: Effects of music-based interventions on cancer-related pain, fatigue, and distress: an overview of systematic reviews
Source: Support Care Cancer. 2023 Jul 24;31(8):488. doi: 10.1007/s00520-023-07938-6 (PMC10366242; doi:10.1007/s00520-023-07938-6)
Supplement: Supplementary file 2 — Supplementary file2 (DOCX 36 KB) [file 520_2023_7938_MOESM2_ESM.docx]

**Supplementary File B.** Excluded records from the umbrella after full text reading (n = 29)

**Reason 1: Meta-analyses included study designs other than randomized controlled trials (n = 1)**

1. Sheikh-Wu, Kauffman MA, Anglade D, Shamsaldeen F, Ahn S, Downs CA. Effectiveness of different music interventions on managing symptoms in cancer survivors: A meta-analysis. *Eur J Oncol Nurs*. 2021; 52: 101968.

<https://doi.org/10.1016/j.ejon.2021.101968>

**Reason 2: Meta-analyses did not evaluate the intervention of interest (n = 8)**

1. Boehm K, Cramer H, Staroszynski T, Ostermann T. Arts therapies for anxiety, depression, and quality of life in breast cancer patients: a systematic review and meta-analysis. *Evid Based Complement Alternat Med*. 2014; 2014: 103297.

<https://doi.org/10.1155/2014/103297>

1. Cheng P, Xu L, Zhang J, Liu W, Zhu J. Role of arts therapy in patients with breast and gynecological cancers: a systematic review and meta-analysis. *J Palliat. Med.* 2021; 24(3): 443- 52.

<https://doi.org/10.1089/jpm.2020.0468>

1. Matthews H, Grunfeld EA, Turner A. The efficacy of interventions to improve psychosocial outcomes following surgical treatment for breast cancer: a systematic review and meta-analysis. *Psycho-Oncology.* 2017; 26(5): 593–607.

<https://doi.org/10.1002/pon.4199>

1. Ruano A, García-Torres F, Gálvez-Lara M, Moriana JA. Psychological and non-pharmacologic treatments for pain in cancer patients: a systematic review and meta-analysis. *J Pain Symptom Manage*. 2022; 63(5): e505–20

<https://doi.org/10.1016/j.jpainsymman.2021.12.021>

1. Tao W, Luo X, Cui B, Liang D, Wang C, Duan Y, et al. Practice of traditional Chinese medicine for psycho-behavioral intervention improves quality of life in cancer patients: A systematic review and meta-analysis. *Oncotarget.* 2015; 6(37): 39725-39.

<https://doi.org/10.18632/oncotarget.5388>

1. Warth M, Kessler J, Koehler F, Aguilar-Raab C, Bardenheuer HJ, Ditzen B. Brief psychosocial interventions improve quality of life of patients receiving palliative care: A systematic review and meta-analysis. *Palliat Med*. 2019; 33(3): 332-45.

<https://doi.org/10.1177/0269216318818011>

1. Warth M, Zöller J, Köhler F, Aguilar-Raab C, Keller J, Ditzen B. Psychosocial interventions for pain management in advanced cancer patients: a systematic review and meta-analysis. *Current Oncol Rep*. 2020; 22(1):3.

<https://doi.org/10.1007/s11912-020-0870-7>

1. Zhao X-X, Cui M, Geng Y-H, Yang Y-L. A systematic review and meta-analysis of randomized controlled trials of palliative care for pain among Chinese adults with cancer. BMC Palliat Care. 2019;18(1).

http://dx.doi.org/10.1186/s12904-019-0456-z

**Reason 3: Medical condition of interest was not meta-analysed alone (n = 3)**

1. Lee JH. The effects of music on pain: A meta-analysis. *J Music Ther.* 2016; 53(4): 430-77.

<https://doi.org/10.1093/jmt/thw012>

1. Dileo C. Effects of music and music therapy on medical patients: A meta-analysis of the research and implications for the future. *J Soc Integr Oncol.* 2006; 4(2): 67-70. doi: 10.2310/7200.2006.002.

<https://europepmc.org/article/med/19442338>

1. Köhler F, Martin ZS, Hertrampf RS, Gäbel C, Kessler J, Ditzen B, et al. Music therapy in the psychosocial treatment of adult cancer patients: A systematic review and meta-analysis. *Front in Psychol.* 2020; 11: 651.

<https://doi.org/10.3389/fpsyg.2020.00651>

**Reason 4: Language of publication other than Spanish or English (n = 3)**

1. Hung T, Liu YC, Tsai PC, Lin MF. The pain-relief efficacy of passive music-based interventions in cancer patients undergoing diagnostic biopsies and surgery: A systematic review and meta-analysis. *Hu Li Za Zhi.* 2018; 65(1): 70-82.

<https://doi.org/>[10.6224/JN.201802_65(1).10](https://doi.org/10.6224/jn.201802_65(1).10)

1. Chae JH, Kim YS, Han MY. Effects of Non-pharmacological Interventions on Cancer Patients with Sleep Disorder: A Meta-analysis. Asian Oncol Nurs. 2021;21(1):1-14.

<http://doi.org/10.5388/aon.2021.21.1.1>

1. Min YC, Oh PJ.   A Meta-Analysis of Intervention Studies on Cancer Pain.   J Korean Oncol Nurs. 2011 Feb;11(1):83-92.

<https://doi.org/10.5388/jkon.2011.11.1.83>

**Reason 5: Meta-analyses included adult and children population (n = 2)**

1. Li Y, Xing X, Shi X, Yan P, Chen Y, Li M, et al. The effectiveness of music therapy for patients with cancer: A systematic review and meta-analysis. *J Adv Nurs*. 2020; 76(5): 1111–23.

<https://doi.org/>10.1111/jan.14313

1. Zhang JM, Wang P, Yao J, Zhao L, Davis MP, Walsh D, et al. Music interventions for psychological and physical outcomes in cancer: A systematic review and meta-analysis. *Support Care Cancer.* 2012; 20(12): 3043-53.

<https://doi.org/>10.1007/s00520-012-1606-5

**Reason 6: Systematic review without meta-analysis (n = 9)**

1. Archer S, Buxton S, Sheffield D. The effect of creative psychological interventions on psychological outcomes for adult cancer patients: a systematic review of randomised controlled trials. *Psychooncology.* 2015; 24(1): 1-10.

<https://doi.org/>10.1002/pon.3607

1. Bardia A, Barton DL, Prokop LJ, Bauer BA, Moynihan TJ. Efficacy of complementary and alternative medicine therapies in relieving cancer pain: a systematic review. *J Clin Oncol*. 2006; 24(34): 5457-64.

<https://doi.org/10.1200/JCO.2006.08.3725>

1. Rennie C, Irvine DS, Huang E, Huang J. Music therapy as a form of nonpharmacologic pain modulation in patients with cancer: a systematic review of the current literature. *Cancers (Basel).* 2022; 14(18): 4416*.*

<https://doi.org/10.3390/cancers14184416>

1. Sundaramurthi T, Gallagher N, Sterling B. Share. Cancer-related acute pain: a systematic review of evidence-based interventions for putting evidence into practice. *Clin J Oncol Nurs.* 2017; 21(3 Suppl): 13-30.

<https://doi.org/10.1188/17.CJON.S3.13-30>

1. Teo I, Krishnan A, Lee GL. Psychosocial interventions for advanced cancer patients: A systematic review*. Psychooncology.* 2019; 28(7): 1394-1407.

<https://doi.org/10.1002/pon.5103>

1. Tola YO, Chow KM, Liang W. Music therapy as a form of nonpharmacologic pain modulation. Effects of non-pharmacological interventions on preoperative anxiety and postoperative pain in patients undergoing breast cancer surgery: A systematic review. *J Clin Nurs.* 2021; 30(23-24): 3369-3384.

[https://doi.org/10.1111/jocn.15827](https://doi.org/10.3390/cancers14184416)

1. Archie P, Bruera E, Cohen L. Music-based interventions in palliative cancer care: a review of quantitative studies and neurobiological literature. Support Care Cancer. 2013;21(9):2609–24.

<http://dx.doi.org/10.1007/s00520-013-1841-4>

1. Yuan D, Huang Y, Wu J, Guo Z, Li S, Zhang Y. Anxiety and depression in lung cancer: effect of psychological interventions - network meta-analysis. BMJ Support Palliat Care. 2022;

<http://dx.doi.org/10.1136/spcare-2022-003808>

1. Yuan D, Huang Y, Wu J, Guo Z, Li S, Zhang Y. Anxiety and depression in lung cancer: effect of psychological interventions - network meta-analysis. BMJ Support Palliat Care. 2022.

http://dx.doi.org/10.1136/spcare-2022-003808

**Reason 7: Congress proceedings (n = 2)**

1. Chen HH. The effect of music intervention in cancer patients receiving radiation therapy: a systematic review and meta-analysis. *Int J Radiat Oncol Biol Phys.* 2020; 108(3): e223.

<https://doi.org/10.1016/j.ijrobp.2020.07.1486>

1. Duhovska J, Baltina D, Millere I, Martinsone K. Effectiveness of music therapy on psychosocial outcomes in patients with cancer experience: systematic review with meta-analysis. *Nordic J Music Ther.* 2016;2 5(S1): 126.

<https://doi.org/10.1080/08098131.2016.1180156>

**Reason 8: Study design other than a systematic review (n = 1)**

1. Chow R, Bergner R, Prisc E. Music therapy effectiveness by duration in patients with cancer: a meta-regression. *BMJ Support & Palliat Care.* 2021; Online First. 18 Jube 2021

<https://doi.org/10.1136/bmjspcare-2021-003163>
