# Supplementary material for: Effects of music-based interventions on cancer-related pain, fatigue, and distress: an overview of systematic reviews
Source: Support Care Cancer. 2023 Jul 24;31(8):488. doi: 10.1007/s00520-023-07938-6 (PMC10366242; doi:10.1007/s00520-023-07938-6)
Supplement: Supplementary file 3 — Supplementary file3 (DOCX 15 KB) [file 520_2023_7938_MOESM3_ESM.docx]

**Supplementary File C.** Presence of spin in the abstracts of included systematic reviews (n = 13)

| Items | Yes  n (%) | No  n (%) |
| --- | --- | --- |
| Item 1: The conclusion formulates recommendations for practice not supported by findings | 3 (23.1%) | 10 (76.9%) |
| Item 2: The title claims or suggests a beneficial effect of the experimental group not supported by findings | 4 (30.8%) | 9 (69.2%) |
| Item 3: Selective reporting of or overemphasis on efficacy or analysis favoring the beneficial effect of the experimental group | 5 (38.5%) | 8 (61.5%) |
| Item 4: The conclusion claims safety based on non-statistically significant results with a wide confidence interval | 0 (0%) | 13 (100%) |
| Item 5: The conclusion claims a beneficial effect of the experimental group despite high risk of bias in primary studies | 7 (53.9%) | 6 (46.1%) |
| Item 6: Selective reporting of or overemphasis on harm outcomes or analysis favoring the safety of the experimental group | 0 (0%) | 13 (100%) |
| Item 7: The conclusion extrapolates the findings to a different intervention | 2 (15.4%) | 11 (84.6%) |
