# Supplementary material for: Effects of music-based interventions on cancer-related pain, fatigue, and distress: an overview of systematic reviews
Source: Support Care Cancer. 2023 Jul 24;31(8):488. doi: 10.1007/s00520-023-07938-6 (PMC10366242; doi:10.1007/s00520-023-07938-6)
Supplement: Supplementary file 4 — Supplementary file4 (DOCX 52 KB) [file 520_2023_7938_MOESM4_ESM.docx]

**Supplementary file D**. Citation Matrix and corrected covered area (CCA) for music-based interventions in cancer

| Randomized controlled trials | Reviews |
| --- | --- |
| 1. Alam M, et al. Utility of recorded guided imagery and relaxing music in reducing patient pain and anxiety, and surgeon anxiety, during cutaneous surgical procedures: A single-blinded randomized controlled trial. *J Am Acad Dermatol.* 2016; 75(3): 585-589. | - Bradt et al. 2021 |
| 2. Alcântara-Silva TR, et al. Music therapy reduces radiotherapy-induced fatigue in patients with breast or gynecological cancer: A randomized trial. *Integr Cancer Ther.* 2018; 17(3): 628-635. | - Bradt et al. 2021 |
| 3. Arruda MALB, Garcia MA, Garcia JBS. Evaluation of the effects of music and poetry in oncologic pain relief: A randomized clinical trial. *J Palliat Med.* 2016; 19(9): 943-948. | - Bradt et al. 2021 - Yangöz et al.2019 |
| 4. Bates D, et al. Music therapy for symptom management after autologous stem cell transplantation: Results from a randomized study. *Biol Blood Marrow Transplant.* 2017; 23(9): 1567-1572. | - Bradt et al. 2021 - Sezgin et al. 2022 |
| 5. Beck SLC. The therapeutic use of music for cancer-related pain. *Oncol Nurs Forum* 1991; 18(8): 1327-1337. | - Bradt et al. 2021 |
| 6. Bieligmeyer S, et al. Feeling the sound - short-term effect of a vibroacoustic music intervention on well-being and subjectively assessed warmth distribution in cancer patients. A randomized controlled trial. *Complement Ther Med.* 2018; 40: 171-178. | - Bradt et al. 2021 |
| 7. Bilgiç Ş and Acaroğlu R. Effects of listening to music on the comfort of chemotherapy patients. *West J Nurs Res.* 2017; 39(6): 745-762. | - Yangöz et al.2019 |
| 8. Binns‐Turner PG, et al. Perioperative music and its effect on anxiety, hemodynamics, and pain in women undergoing mastectomy. *ANNA J.* 2011; 79(4Suppl): S21-S27. | - Bradt et al. 2021 - Bro et al. 2018 - Nightingale et al. 2013 - Wang et al. 2018 |
| 9. Bradt J, et al. The impact of music therapy versus music medicine on psychological outcomes and pain in cancer patients: a mixed methods study. *Support Care Cancer.* 2015; 23(5): 1261-1271. | - Bradt et al. 2021 - Yangöz et al. 2019 |
| 10. Bro ML, et al. Effects of live music during chemotherapy in lymphoma patients: a randomized, controlled, multi-center trial. *Support Care Cancer.* 2019; 27(10): 3887-3896. | - Bradt et al. 2021 - Nguyen et al. 2022 |
| 11. Bulfone T, et al. Effectiveness of music therapy for anxiety reduction in women with breast cancer in chemotherapy treatment. *Holist Nurs Pract.* 2009; 23(4): 238‐242. | - Bradt et al. 2021 - Bro et al. 2018 - Nguyen et al. 2022 - Nightingale et al. 2013 - Tsai et al.2014 - Wang et al. 2018 |
| 12. Burns DS. The effect of the Bonny method of guided imagery and music on the mood and life quality of cancer patients. *J Music Ther.* 2001; 38(1): 51-65. | - Bradt et al. 2021 |
| 13. Burns DS, et al. Music imagery for adults with acute leukemia in protective environments: a feasibility study. *Support Care Cancer.* 2008; 16(5): 507-513. | - Bro et al. 2018 - Nguyen et al. 2022 - Nightingale et al. 2013 - Sezgin et al. 2022 - Tsai et al. 2014 |
| 14. Burns DS, et al. Differences between supportive music and imagery and music listening during outpatient chemotherapy and potential moderators of treatment effects. *J Music Ther.* 2018; 55(1): 83-108. | - Bradt et al. 2021 |
| 15. Burrai F, et al. Effects of live sax music on various physiological parameters, pain level, and mood level in cancer patients. *Holist Nurs Pract.* 2014; 28(5): 301‐311. | - Bradt et al. 2021 - Bro et al. 2018 - Garza-Villarreal et al. 2017 - Park et al. 2021 - Yangöz et al. 2019 |
| 16. Cai GR, Li PW, Jiao LP. Clinical observation of music therapy combined with anti-tumor drugs in treating 116 cases of tumor patients. *Zhongguo Zhong Xi Yi Jie He Za Zhi*. 2001; 21(12): 891-894. | - Bradt et al. 2021 |
| 17. Cassileth BR, et al. Music therapy for mood disturbance during hospitalization for autologous stem cell transplantation. *Cancer.* 2003; 98: 2723-2729. | - Bradt et al. 2021 - Bro et al. 2018 - Nightingale et al. 2013 - Qi et al. 2021 - Sezgin et al. 2022 - Tsai et al. 2014 |
| 18. Chen L, et al. The effect of cognitive behavioral therapy on quality of life in patients with breast cancer. *Chin J Clin Rehabil*. 2002; 6: 2686-2687. | - Wang et al. 2018 |
| 19. Chen L, et al. Effect of cognitive behavioral intervention therapy on immunological function of patients with breast cancer. *Chin J Clin Rehabil.* 2004; 8: 6310-6311. | - Bradt et al. 2021 - Wang et al. 2018 |
| 20. Chen LC, et al. Fifteen-minute music intervention reduces pre-radiotherapy anxiety in oncology patients. *Eur J Oncol Nurs*. 2013; 17(4): 436-441. | - Bradt et al. 2021 |
| 21. Chen SC, et al. Comparison of group vs self-directed music interventions to reduce chemotherapyrelated distress and cognitive appraisal: an exploratory study. *Supportive Care Cancer*. 2018; 26(2): 461-469. | - Bradt et al. 2021 |
| 22. Chen SC, et al. Music, heart rate variability, and symptom clusters: a comparative study. *Support Care Cancer.* 2020; 28(1): 351-360. | - Bradt et al. 2021 - Nguyen et al. 2022 - Qi et al 2021 |
| 23. Chirico A, et al. Virtual reality and music therapy as distraction interventions to alleviate anxiety and improve mood states in breast cancer patients during chemotherapy. *J Cell Physiol*. 2020; 235(6): 5353-5362. | - Qi et al 2021 |
| 24. Clark M, et al. Use of preferred music to reduce emotional distress and symptom activity during radiation therapy. *J Music Ther.* 2006; 43(3): 247‐265. | - Bradt et al. 2021 - Bro et al 2018 - Garza-Villarreal et al. 2017 - Nightingale et al. 2013 - Tsai et al. 2014 |
| 25. Cook EL, Silverman MJ. Effects of music therapy on spirituality with patients on a medical oncology / hematology unit: a mixed‐methods approach. *Arts Psychother.* 2013; 40: 239‐244. | - Bradt et al. 2021 - Bro et al. 2018 |
| 26. Danhauer SC, et al. Music for patients with hematological malignancies undergoing bone marrow biopsy: a randomized controlled study of anxiety, perceived pain, and patient satisfaction. *J Soc Integr Oncol.* 2010; 8: 140-147. | - Bradt et al. 2021 - Nightingale et al. 2013 |
| 27. Doro CA, et al. Music therapy improves the mood of patients undergoing hematopoietic stem cells transplantation (controlled randomized study). *Support Care Cancer.* 2017; 25(3): 1013-1018. | - Bradt et al. 2021 |
| 28. Ferrer AJ. The effect of live music on decreasing anxiety in patients undergoing chemotherapy treatment. J *Music Ther*. 2007; 44(3): 242-255. | - Bradt et al. 2021 - Bro et al. 2018 - Nguyen et al. 2022 - Nightingale et al. 2013 - Qi et al. 2021 - Tsai et al. 2014 |
| 29. Firmeza MA, et al. Control of anxiety through music in a head and neck outpatient clinic: a randomized clinical trial. *Rev Esc de Enferm USP*. 2017; 51: e03201. | - Bradt et al. 2021 |
| 30. Fredenburg HA, Silverman MJ. Effects of music therapy on positive and negative affect and pain with hospitalized patients recovering from a blood and marrow transplant: a randomized effectiveness study. *Arts Psychother.* 2014; 41: 174‐180. | - Bradt et al. 2021 - Bro et al. 2018 |
| 31. Fu Y, et al. Investigation on the application of music therapy to improve the anxiety and depression of advanced breast cancer chemotherapy with the FEC. *Chin J Misdiagnostics* 2009; 9: 8312-8313. | - Wang et al. 2018 |
| 32. Gao J, et al. Analysis of the influence of five-element music on the psychological negative emotion of leukemia patients undergoing chemotherapy. *Front Med.* 2020;10(7): 209–210. | - Yang et al. 2021 |
| 33. Gimeno M. The effect of music and imagery to induce relaxation and reduce nausea and emesis in cancer patients undergoing chemotherapy treatment [PhD thesis]. Stockton, CA: University of the Pacific, 2008. | - Bradt et al. 2021 |
| 34. Gu H. Effect of yoga music on anxiety and pain of patients with breast cancer after PICC operation. *Today Nurs.* 2015; 71–72 | - Wang et al. 2018 |
| 35. Hanser SB, et al. Effects of a music therapy intervention on quality of life and distress in women with metastatic breast cancer. *J Soc Integrative Oncology.* 2006; 4(3): 116‐124. | - Bradt et al. 2021 - Bro et al. 2018 - Nightingale et al. 2013 - Tsai et al. 2014 - Wang et al. 2018 |
| 36. Harper EI. Reducing treatment-related anxiety in cancer patients: comparison of psychological interventions [dissertation]. Dallas, TX: Southern Methodist University; 2001 | - Bradt et al. 2021 - Nightingale et al. 2013 |
| 37. Herbrand MK, Silverman MJ. A randomized pilot study of patient-preferred live music addressing fatigue, energy, and pain in adults on a medical oncology/hematology unit. *Psychol Music.* 2021; 49(6): 1561-1572. | - Sezgin et al. 2022 |
| 38. Hilliard RE. The effects of music therapy on the quality and length of life of people diagnosed with terminal cancer. *J Music Ther.* 2003; 40(2): 113-137. | - Bradt et al. 2021 |
| 39. Huang ST, et al. The effectiveness of music in relieving pain in cancer patients: a randomised controlled trial. *Int J Nurs Stud.* 2010; 47(11): 1354-1362. | - Bradt et al. 2021 - Bro et al. 2018 - Garza-Villarreal et al 2017 - Park et al. 2021 - Tsai et al. 2014 - Yangöz et al. 2021 |
| 40. Huang Y, Yang X, Yang Q. Effect of traditional Chinese medicine five elements music on cancer-related fatigue of chemotherapy patients. *Chin J Mod Nurs.* 2012; 18: 1412-1414. | - Tao et al. 2016 - Yang et al. 2021 |
| 41. Huang Y. Effect of music intervention on the depression mental of post-operative chemotherapy interval patients with breast cancer. *Chin J Mod Nurs.* 2012;18: 552–554 | - Wang et al. 2018 |
| 42. Hunter JJ, et al. A randomized trial of nurse-administered behavioral interventions to manage anticipatory nausea and vomiting in chemotherapy. *Cancer Medicine* 2020; 9(5): 1733-1740. | - Bradt et al. 2021 |
| 43. Gao J, et al. Analysis of the influence of five-element music on the psychological negative emotion of leukemia patients undergoing chemotherapy. *Front Med.* 2020;10(7): 209–210. | - Yang et al. 2021 |
| 44. Jasemi M, et al. The effects of music therapy on anxiety and depression of cancer patients. *Indian J Palliat Care*. 2016 ;22(4): 455-458. | - Bradt et al. 2021 |
| 45. Jin Y, et al. Effects of music assisted therapy on relieving chemotherapy-induced adverse effects of patients with breast cancer operation. *Nurs J Chin PLA.* 2008; 25: 1-3. | - Wang et al. 2018 |
| 46. Jin F, Zhao Y. Influence of music relaxation therapy on vital signs and anxiety of liver cancer patients accepting transcatheter hepatic arterial chemoembolization*. Huli Yanjiu.* 2011; 16: 1429-1431. | - Bradt et al. 2021 |
| 47. Karadag E. The effect of music listening intervention applied during radiation therapy on the anxiety and comfort level in women with early-stage breast cancer: a randomized controlled trial. *Eur J Integrat Med.* 2019; 27: 39-44. | - Bradt et al. 2021 |
| 48. Keenan A. Effect of a music intervention on pain following hematopoietic stem cell transplant [PhD thesis]. Rush University, 2017 | - Bradt et al. 2021 |
| 49. Kwekkeboom KL. Music versus distraction for procedural pain and anxiety in patients with cancer. *Oncol Nurs Forum.* 2003; 30: 433-440. | - Bradt et al. 2021 - Nightingale et al. 2013 |
| 50. Lei YY. Effect of five elements music of traditional Chinese medicine on the tumor patients under hyperthermia nursing. *J Nurses Train.* 2014; 29: 335-336. | - Tao et al. 2016 |
| 51. Letwin L, Silverman MJ. No between-group differences but tendencies for patient support: a pilot study of a resilience-focused music therapy protocol for adults on a medical oncology/hematology unit. *Arts Psychother.* 2017; 55: 116-125. | - Bradt et al. 2021 |
| 52. Li S. Applying Chinese classical music to treat preoperative anxiety of patients with gastric cancer. *Huli Yanjiu* 2004; 18(3B): 471-472. | - Bradt et al. 2021 - Tsai et al. 2014 |
| 53. Li XM, Yan H, Zhou KN, Dang SN, Wang DL, Zhang YP. Effects of music therapy on pain among female breast cancer patients after radical mastectomy: results from a randomized controlled trial. *Breast Cancer Res Treat.* 2011; 128: 411‐419. | - Bro et al. 2018 - Garza-Villarreal 2013 - Park et al. 2021 - Tsai et al. 2014 - Wang et al. 2018 |
| 54. Li XM, et al. Effects of music therapy on anxiety of patients with breast cancer after radical mastectomy: a randomized controlled trial. *J Adv Nurs.* 2011; 68(5): 1145‐1155. | - Bradt et al. 2021 - Bro et al. 2018 |
| 55. Liang Y, Jiang X. Effect of music therapy on patients with breast cancer during perioperative period. *Chin Community Doct*. 2010; 12: 46–47. | - Wang et al. 2018 |
| 56. Liao J, et al. Effects of Chinese medicine five-element music on the quality of life for advanced cancer patients: a randomized controlled trial. *Chin J Integr Med.* 2013;19(10): 736-740. | - Bradt et al. 2021 - Yang et al. 2021 |
| 57. Lima TU, et al. Impact of a music intervention on quality of life in breast cancer patients undergoing chemotherapy: a randomized clinical trial. *Integr Cancer Ther*. 2020; 19: 1534735420938430 | - Nguyen et al. 2022 |
| 58. Lin MF, et al. A randomized controlled trial of the effect of music of music therapy and verbal relaxation on chemotherapy‐induced anxiety. *J Clin Nurs*. 2011; 20: 988‐999. | - Bradt et al. 2021 - Bro et al. 2018 - Nguyen et al. 2022 - Nightingale et al. 2013 - Tsai et al. 2014 |
| 59. Liu XF, Liu XL. Effect of five-element music intervention on depression and sleep quality of lung cancer patients, *Chin Med Modern Distance Ed China.* 2019; 17(11): 50-52. | - Yang et al. 2021 |
| 60. Lu Z, Hu Y. The effect of music relaxation therapy on the adverse reactions induced by chemotherapy in patients with breast cancer. *Chin J Nurs.* 2010;45: 405-408. | - Wang et al. 2018 |
| 61. Lu L. Clinical study of music therapy on improving the symptoms of malignant tumor patients. Hebei Medical University, Shijiazhuang 2019. | - Qi et al. 2021 |
| 62. Lv H, Chen J, Chen J, Peng S, Yang X: Effect of personalized music intervention on the preoperative anxiety in patients undergoing breast cancer surgery*. J Nurs Sci.* 2008; 23: 4-6. | - Wang et al. 2018 |
| 63. Ma X. Clinical application of playing music in patients with breast cancer resection. *Liuzhou Med.* 2013; 26:17-19. | - Wang et al. 2018 |
| 64. Madden JR, et al. Creative arts therapy improves quality of life for pediatric brain tumor patients receiving outpatient chemotherapy*. J Pediatr Oncol Nurs.* 2010; 27(3): 133-145. | - Tsai et al. 2014 |
| 65. Mische Lawson L, et al. Effects of making art and listening to music on symptoms related to blood and marrow transplantation. *Oncol Nurs Forum.* 2016; 43(2) :56‐63. | - Bro et al. 2018 |
| 66. Mondanaro JF, et al. The effects of clinical music therapy on resiliency in adults undergoing infusion: a randomized, controlled trial. *J Pain Symptom Manag*. 2021; 61(6): 1099-1108. | - Nguyen et al. 2022 |
| 67. Moradian S, et al. Nevasic audio program for the prevention of chemotherapy induced nausea and vomiting: a feasibility study using a randomised controlled trial design. *Eur J Oncol Nurs.* 2015; 19: 282-291. | - Bradt et al. 2021 - Bro et al. 2018 |
| 68. Mou Q, et al. Effects of passive music therapy on anxiety and vital signs in lung cancer patients undergoing peripherally inserted central catheter placement procedure. *J Vascular Access* 2020; 21(6): 875-882. | - Bradt et al. 2021 |
| 69. O'Callaghan C, et al. Effect of self-selected music on adults' anxiety and subjective experiences during initial radiotherapy treatment: a randomised controlled trial and qualitative research. *J Med Imaging Radiat Oncol.* 2012; 56(4): 473-477. | - Bradt et al. 2021 - Bro et al. 2018 |
| 70. Palmer J, et al. Effects of music therapy on anaesthesia requirements and anxiety in women undergoing ambulatory breast surgery for cancer diagnosis and treatment: a randomised controlled trial. *J Clin Oncol.* 2015; 33(28): 3162-3168. | - Bradt et al. 2021 |
| 71. Pan YN, et al. Analysis of the effect of traditional Chinese medicine five-tone therapy on depression of patients with digestive system tumor during chemotherapy. *Hebei Medical Journal* 2017; 39(14): 2211-2213. | - Yang et al. 2021 |
| 72. Pan Q, et al. Effect of individualized music therapy on gastrointestinal reaction in patients with breast cancer *Hainan Med J.* 2015; 26: 1553-1554. | - Wang et al. 2018 |
| 73. Pan YQ, et al. Effect of Zhi tone therapy on anxiety and depression in 30 cancer patients undergoing chemotherapy, *Fujian J Trad Chin Med*. 2019; 50(6): 87-88. | - Yang et al. 2021 |
| 74. Pedersen MRV, et al. Music and pain during endorectal ultrasonography examination: a prospective questionnaire study and literature review. *Radiography* 2020; 26(3): E164-E169. | - Bradt et al. 2021 |
| 75. Pinto Junior FEL, et al. Influence of music on pain and anxiety due to surgery in patients with breast cancer *Rev Bras Cancerol.* 2012; 58(2): 135-141. | - Bradt et al. 2021 |
| 76. Ratcliff CG, et al. Music therapy for patients who have undergone hematopoetic stem cell transplant. *Evid Based Complement Alternat Med.* 2014; 2014: 742941 | - Bradt et al. 2021 - Bro et al. 2018 |
| 77. Reimnitz L, Silverman MJ. A randomized pilot study of music therapy in the form of patient-preferred live music on fatigue, energy, and pain in hospitalized adult oncology patients on a blood and marrow transplant unit. *Arts & Health* 2020; 12(2): 154-168. | - Bradt et al. 2021 - Qi et al. 2018 - Sezgin et al. 2022 |
| 78. Ren X, et al. Effects of personalized music intervention depression in patients of breast cancer during chemotherapy period. *Chin J Mod Med.* 2010; 20: 771–774. | - Wang et al. 2018 |
| 79. Romito F, et al. Music therapy and emotional expression during chemotherapy. How do breast cancer patients feel? *Eur J Integr Med*. 2013; 5(5): 438-442. | - Bradt et al. 2021 |
| 80. Rosenow SC, Silverman MJ. Effects of single session music therapy on hospitalized patients recovering from a bone marrow transplant: two studies. *Arts Psychother.* 2014; 41(1): 65-70. | - Bradt et al. 2021 - Bro et al. 2018 - Qi et al. 2021 - Sezgin et al. 2022 |
| 81. Rossetti A, et al. The impact of music therapy on anxiety in cancer patients undergoing simulation for radiation therapy. *Int J Radiat Oncol Biol Phys.* 2017; 99(1): 103-110. | - Bradt et al. 2021 |
| 82. Shaban M, et al. Study of two non-pharmacological methods, progressive muscle relaxation and music on pain relief of cancerous patients. *J Tehran Fac Nurs & Midwifery* 2006; 12(3): 87. | - Bradt et al. 2021 |
| 83. Smith M, et al. Music as a therapeutic intervention for anxiety in patients receiving radiation therapy. *Oncol Nurs Forum.* 2001; 28(5): 855‐862. | - Bradt et al. 2021 - Bro et al. 2018 - Nightingale et al. 2013 |
| 84. Stordahl JJ. *The influence of music on depression, affect, and benefit finding among women at the completion of treatment for breast cancer* [PhD thesis]. Miami, FL: University of Miami, 2009 | - Bradt et al. 2021 |
| 85. Straw GW. The use of guided imagery and relaxation for the quality of life of cancer patients undergoing chemotherapy [Master's thesis]. Ontario: Lakehead University, 1991. | - Bradt et al. 2021 |
| 86. Sun W, et al. Effects of personalized music intervention on reducing adverse psychological reaction in patients with breast cancer during chemotherapy. *Anhui Med Pharm J* 2013; 17: 1441-1443. | - Wang et al. 2018 |
| 87. Toccafondi A, et al. Live music intervention for cancer inpatients: The Music Givers format. *Palliat Support Care.* 2018; 16(6): 777-784. | - Yangöz et al. 2019 |
| 88. Tuinmann G, et al. The effects of music therapy in patients with high-dose chemotherapy and stem cell support: a randomized pilot study. *Psychooncology.* 2017; 26(3): 377-384. | - Bradt et al. 2021 - Nguyen et al. 2022 |
| 89. Vachiramon V, et al. Music reduces patient anxiety during Mohs surgery: an openlabel randomized controlled trial. Dermatol Surg. 2013; 39(2): 298-305. | - Bradt et al. 2021 - Bro et al. 2018 |
| 90. Verstegen AL, Silverman MJ. Effects of hope-based music therapy on hope and pain in hospitalized patients on blood and marrow transplant unit: a convergent parallel mixed methods pilot study [MA Thesis]. Ann Arbor: University of Minnesota, 2016. | - Bradt et al. 2021 |
| 91. Verstegen AL, Silverman MJ. Effects of music therapy on mood and pain with patients hospitalized for bone marrow transplantation: a randomized effectiveness pilot study. *J Creat Ment Health* 2018; 13(4): 418-428. | - Bradt et al. 2021 |
| 92. Wan Y, et al. Influence of music therapy on anxiety, depression, and pain of cancer patients. *Huli Yanjiu* 2009; 23(5A): 1172-5. | - Bradt et al. 2021 |
| 93. Wang Y. Application of music therapy in patients with breast cancer during chemotherapy. *J Community Med.* 2009; 7: 26-27. | - Wang et al. 2018 |
| 94. Wang H, Guo Y. Effects of post-operative music therapy on reducing vomiting in patients with breast cancer during chemotherapy. *J Changzhi Med Coll.* 2011; 25: 392-393. | - Wang et al. 2018 |
| 95. Wang Y, et al. Effects of intravenous patient-controlled sufentanil analgesia and music therapy on pain and haemodynamics aSer surgery for lung cancer: a randomised parallel study. *J Altern Complement Med* 2015; 21(11): 667-672. | - Bradt et al. 2021 |
| 96. Wang XY. The improvement of progressive muscle relaxation training combined with traditional Chinese medical music on anxiety depression and quality of life of cancer patients. *J Mod Oncol* 2017; 25: 3509-3512. | - Qi et al. 2021 |
| 97. Wang W. Effect of five-tone therapy on stress state of breast cancer patients during chemotherapy. *Clin J Chin Med*. 2019;11(7): 66–68. | - Yang et al. 2021 |
| 98. Wen MH, et al. Effect of traditional Chinese medicine five-tone therapy on quality of life of patients with gynecological malignant tumor during chemotherapy. *J New Chin Med*. 2016; 48(1): 160-161. | - Yang et al. 2021 |
| 99. Wren AA, et al. Preliminary efficacy of a loving kindness meditation intervention for patients undergoing biopsy and breast cancer surgery: a randomized controlled pilot study. *Supportive Care Cancer* 2019; 27(9): 3583-3592. | - Bradt et al. 2021 |
| 100. Wu Y. Psychological intervention and influence of background music in modified radical mastectomy for breast cancer. *Heilongjiang Med J* 2009; 33: 709-710. | - Wang et al. 2018 |
| 101. Wu Y, et al. Effects of five-tone music therapy on reducing pre-operative anxiety in patients with breast cancer *Fujian Med J* 2014; 36: 148-149. | - Wang et al. 2018 |
| 102. Xie Z, et al. Effect of music therapy and inner image relaxation on quality of life in cancer patients receiving chemotherapy. *Zhongguo Xinli Weisheng Zazhi* 2001; 15(3): 176-178. | - Bradt et al. 2021 |
| 103. Xie J. Effects of music therapy on patients with advanced breast cancer during chemotherapy. *Today Nurs.* 2006; 76-77. | - Wang et al. 2018 |
| 104. Xu L, Wang Y. Application of background music in modified radical mastectomy for breast cancer. *Chin Mod Doct.* 2010; 48: 75-76. | - Wang et al. 2018 |
| 105. Xu HY, et al. Effect of five-element music therapy on negative psychology and sleep quality of patients with advanced cancer. *J. Tradit Chin Med.* 2019; 60(11): 954-956. | - Yang et al. 2021 |
| 106. Yahui F, et al. Effect of five-element music on psychological adjustment of patients with gynecological malignant tumor. *Modern Trad Chin Med*. 2017; 37(5): 36-37. | - Yang et al. 2021 |
| 107. Yang J, et al. Effect of Chinese medicine five-element music intervention on depression of breast cancer patients undergoing chemotherapy. *Chin J Modern Nurs*. 2013; 19(36): 4461-4463. | - Tao et al. 2016 - Wang et al. 2018 - Yang et al. 2021 |
| 108. Yao X, Chen M. Effects of angle mode music on nausea and vomiting in patients with breast cancer during chemotherapy period. *Nei Mongol J Tradit Chin Med*. 2015: 6 | - Wang et al. 2018 |
| 109. Yates GJ, Silverman MJ. Immediate effects of single-session music therapy on aective state inpatients on a post-surgical oncology unit: a randomised effectiveness study. *Arts Psychother.* 2015; 44: 57-61. | - Bradt et al. 2021 - Bro et al. 2018 |
| 110. Zengin S, et al. Effects of music therapy on pain and anxiety in patients undergoing port catheter placement procedure. *Complement Ther Med.* 2013; 21: 689‐696. | - Bro et al. 2018 |
| 111. Zhai W, Liu N. Application effect of music therapy of traditional Chinese medicine in hospice care ward for patients with gastric cancer, *World Chin J Dig*. 2017; 25(4): 388-391. | - Yang et al. 2021 |
| 112. Zhang J, et al. Effects of music therapy on gastrointestinal reaction in patients with breast cancer. *J Med Theory Pract.* 2016; 29:689-691. | - Wang et al. 2018 |
| 113. Zhang LD, et al. Clinical application of five-element music therapy in improving anxiety and depression symptoms of patients with advanced cancer. *Chin Gen Nurs*. 2018;16(30): 3786-3787. | - Yang et al. 2021 |
| 114. Zhao P, et al. Intervention effects of musical therapy to physiological and psychological conditions in process of radiotherapy for patients with cancer. *Chin J Cancer Prev Treat.* 2008; 15: 1097-1099. | - Bradt et al. 2021 - Nightingale et al. 2013 - Tsai et al. 2014 |
| 115. Zheng Z, et al. Effects of point massage in combination with music therapy on post-operative pain in patients with breast cancer. *Chin Pract Med.* 2015; 10: 235-236. | - Wang et al. 2018 |
| 116. Zhou LJ. Study of effects of personalized music intervention on depression and anxiety among breast cancer patients during chemotherapy period. Master’s Thesis, Nursing Department, Central South University, 2009. | - Wang et al. 2018 |
| 117. Zhou K, et al. Effects of music therapy on depression and duration of hospital stay of breast cancer patients after radical mastectomy. *Chin Med J (Engl).* 2011; 124(15): 2321‐2327. | - Bro et al. 2018 - Tsai et al. 2014 - Wang et al 2018 |
| 118. Zhou K, et al. A clinical randomised controlled trial of music therapy and progressive muscle relaxation training in female breast cancer patients after radical mastectomy: results on depression, anxiety and length of hospital stay. *Eur J Oncol Nurs.* 2015; 19: 54-59. | - Bradt et al. 2021 |
| 119. Zimmerman L, et al. Effects of music in patients who had chronic cancer pain. *West J Nurs Res.* 1989; 11(3): 298‐309. | - Bro et al. 2018 |

Music-based interventions in cancer

| CCA = | N-r | = | 202-119 | = | 83 | = 0,0581 = 5.81% |
| --- | --- | --- | --- | --- | --- | --- |
|  | rc-r |  | 1547-119 |  | 1428 |  |

Note: *N* is the number of included publications (including double counting) in the available evidence synthesis (this is the sum of the ticked boxes in the citation matrix); where *r* is the number of rows (number of index publications), and *c* is the number of columns (number of reviews).
