# Supplementary material for: Effects of music-based interventions on cancer-related pain, fatigue, and distress: an overview of systematic reviews
Source: Support Care Cancer. 2023 Jul 24;31(8):488. doi: 10.1007/s00520-023-07938-6 (PMC10366242; doi:10.1007/s00520-023-07938-6)
Supplement: Supplementary file 5 — Supplementary file5 (DOCX 18 KB) [file 520_2023_7938_MOESM5_ESM.docx]

**Supplementary file E**. Graphical Representation of Overlap
